# Impurity of Stem Cell Graft by Murine Embryonic Fibroblasts – Implications for Cell-Based Therapy of the Central Nervous System

**DOI:** 10.3389/fncel.2014.00257

**Published:** 2014-09-05

**Authors:** Marek Molcanyi, Narges Zare Mehrjardi, Ute Schäfer, Nadia Nabil Haj-Yasein, Michael Brockmann, Marina Penner, Peter Riess, Clemens Reinshagen, Bernhard Rieger, Tobias Hannes, Jürgen Hescheler, Bert Bosche

**Affiliations:** ^1^Institute of Neurophysiology, Medical Faculty, University of Cologne, Cologne, Germany; ^2^Clinic of Neurosurgery, Medical Faculty, University of Cologne, Cologne, Germany; ^3^Research Unit for Experimental Neurotraumatology, Medical University of Graz, Graz, Austria; ^4^Department of Nutrition, Institute of Basic Medical Sciences, Faculty of Medicine, University of Oslo, Oslo, Norway; ^5^Department of Pathology, Kliniken der Stadt Köln, Cologne-Merheim Hospital, University of Witten/Herdecke, Cologne, Germany; ^6^Department of Traumatology and Orthopedics, HELIOS Klinik Bad Berleburg, Bad Berleburg, Germany; ^7^Molecular Neurotherapy and Imaging Laboratory, Massachusetts General Hospital, Harvard Medical School, Boston, MA, USA; ^8^Department of Radiology, Massachusetts General Hospital, Harvard Medical School, Boston, MA, USA; ^9^Department of Pediatric Cardiology, Heart Center Cologne, Medical Faculty, University Hospital of Cologne, Cologne, Germany; ^10^Division of Neurosurgery, St Michael’s Hospital, Keenan Research Centre for Biomedical Science and the Li Ka Shing Knowledge Institute of St. Michael’s Hospital, Department of Surgery, University of Toronto, Toronto, ON, Canada; ^11^Department of Neurology, University Hospital of Essen, University of Duisburg-Essen, Essen, Germany

**Keywords:** stem cell transplantation, feeder-based cell line, murine embryonic fibroblasts, stroke, brain injury, cell graft contamination

## Abstract

Stem cells have been demonstrated to possess a therapeutic potential in experimental models of various central nervous system disorders, including stroke. The types of implanted cells appear to play a crucial role. Previously, groups of the stem cell network NRW implemented a feeder-based cell line within the scope of their projects, examining the implantation of stem cells after ischemic stroke and traumatic brain injury. Retrospective evaluation indicated the presence of spindle-shaped cells in several grafts implanted in injured animals, which indicated potential contamination by co-cultured feeder cells (murine embryonic fibroblasts – MEFs). Because feeder-based cell lines have been previously exposed to a justified criticism with regard to contamination by animal glycans, we aimed to evaluate the effects of stem cell/MEF co-transplantation. MEFs accounted for 5.3 ± 2.8% of all cells in the primary FACS-evaluated co-culture. Depending on the culture conditions and subsequent purification procedure, the MEF-fraction ranged from 0.9 to 9.9% of the cell suspensions *in vitro*. MEF survival and related formation of extracellular substances *in vivo* were observed after implantation into the uninjured rat brain. Impurity of the stem cell graft by MEFs interferes with translational strategies, which represents a threat to the potential recipient and may affect the graft microenvironment. The implications of these findings are critically discussed.

## Introduction

Cell replacement strategies have been proposed to be a promising therapeutic approach for various disorders of the central nervous system. Conditions predominantly associated with a loss of one specific cell population, such as amyotrophic lateral sclerosis, Parkinson’s disease, or subarachnoid hemorrhage, may be targeted using specifically pre-differentiated cell grafts. In the case of traumatic or ischemic brain injury, a whole tissue segment (including neurons, glia, and vascular cells) has to be replaced by cells, which are able to differentiate into all lost cell types; alternatively, a heterogeneous graft containing different cell populations can also be used (Schouten et al., 2004; Molcanyi et al., 2007; Riess et al., 2007; Lohr et al., 2008; Burns et al., 2009; Richardson et al., 2010; Benchoua and Onteniente, 2011).

Previously, groups of the Stem Cell Network North-Rhine Westphalia evaluated the use of GFP-positive pluripotent embryonic stem cells (ESCs) after experimental ischemia and traumatic brain injury (TBI). ESC migration and differentiation was reported in ischemic animals (Hoehn et al., 2002; Erdo et al., 2003). In contrast, neurological improvement followed by gradual loss of implanted cells, came to the forefront in TBI-injured rats receiving ESC grafts (Molcanyi et al., 2007, 2013; Riess et al., 2007). Nestin was, *inter alia*, utilized to examine early differentiation along the neural pathway in injured animals. Nestin was co-expressed by only a few GFP-positive ESCs. However, nestin staining was abundant at trauma and implantation sites and was predominantly expressed by cells lacking any co-localization with GFP (e.g., activated resident glia). Additional presence of nestin-expressing, GFP-negative spindle-shaped cellular elements localized inside the implanted graft was observed during confocal analysis of the implanted grafts; however, it was not systematically evaluated in our previous studies (see Supplementary Material) (Molcanyi et al., 2007, 2013). Current re-assessment of previous observations (Molcanyi et al., 2007, 2009) revealed the presence of such cells in several grafted animals. Because implemented ESCs were grown on a feeder layer consisting of inactivated murine embryonic fibroblasts (MEFs), these histological findings raised concerns regarding potential cell graft contamination by co-cultured feeder cells.

In this study, we quantified the amount of MEFs in proportion to co-cultured ESCs *in vitro* under standard conditions and after re-plating procedure. Furthermore, MEF survival was observed *in vivo* after transplantation into healthy rat brain and was evaluated with respect to survival and interaction with the surrounding brain microenvironment. Feeder-based cell lines have been subject to criticism regarding the contamination of ESCs by feeder-derived animal proteins. Our findings revealed the potential of additional graft impurity during the transplantation procedures. The effect of these findings on previously established stem cell protocols is discussed.

## Materials and Methods

### Cell cultures

Murine embryonic fibroblasts cells were prepared from day 13 to 14 embryos (decapitated body, removed inner organs). MEF cells were G418-resistant (selection drug used in isolating homologous recombinants) and thus, prepared from mice harboring the neo gene. We used a CD1 neo mouse, which harbors pSC2neo. MEFs were inactivated using 10-μg/ml mitomycin for 2–3 h prior to culture. For transplantation, the MEF monoculture was trypsinized and resuspended in PBS to achieve a final concentration of 10^3^ cells/μl. For immunohistochemistry, MEFs were cultured on gelatinized coverslips and alternatively on plates in Dulbecco modified Eagle medium (DMEM), containing 10% fetal calf serum (FCS), 1% non-essential amino acids (NEAA), and 50 μM β-mercaptoethanol (all from Thermo Scientific, USA) for further co-culturing with ES cells. The CGR8 feeder-free cell line, which was used as a control cell line for immunohistochemistry, was cultured in GMEM with stable glutamine und sodium pyruvate (Thermo Scientific, Germany) supplemented with 10% FCS, 1000 U/ml leukemia-inhibiting factor (Millipore, Germany), and 50 μM β-mercaptoethanol on coverslips.

Murine ESCs of the D3 cell line stably transfected with the pCX-(-act)-enhanced-GFP expression vector as previously described (Arnhold et al., 2000) were cultured on a feeder-layer in DMEM containing 15% FCS, 1% NEAA, 1% penicillin-streptomycin, 50 μM 2-mercaptoethanol, and 1000 U/ml LIF (Millipore, Germany). ESCs were cultured on plastic dishes in the presence of leukemia-inhibitory factor on a layer of mitotically inactivated MEFs.

### Immunocytochemistry and FACS

Murine embryonic fibroblasts cultured on coverslips were fixed for 5 min in 2% paraformaldehyde, washed twice with PBS, and stained with standard hematoxylin-eosin for morphological evaluation. For immunocytochemistry, the cells were fixed, washed, permeabilized for 15 min in PBS-0.2% Triton X-100, and blocked with 5% normal goat serum (NGS). Incubation with primary antibodies (1:100 dilution in PBS-NGS-Triton solution) was performed for 2 h at room temperature. Rinsing in PBS was followed by incubation with secondary antibodies (1:100, at room temperature for 2 h.) and DAPI-counterstaining. The following primary antibodies were used: anti-mouse nestin (Millipore, Germany) and anti-mouse vimentin (Sigma, USA), anti-mouse-feeder-PE (Miltenyi Biotec, Germany). The following secondary antibody was used: anti-mouse IgG Alexa 555 (Life Technologies, Germany) for nestin und vimentin, and the PE-conjugated anti-feeder antibody signal was amplified using anti-rat IgG Alexa 555 (Life Technologies, Germany). Labeled cells were mounted upside-down onto glass slides with DAKO fluorescent mounting medium (Dako, Denmark) and evaluated using conventional/fluorescent microscopy. Primary antibody was omitted in negative controls. CGR8 was implemented as an additional negative control for anti-mouse-feeder staining to exclude an unspecific binding of the primary antibody.

For FACS analysis, 0.5 × 10^6^ D3-βactin-GFP(P8) ESCs were plated on 0.8 × 10^6^ mitomycin inactivated MEFs. After 2 days, the ESCs were trypsinized or alternatively purified on 0.1% gelatin-coated dishes (Sigma, Germany) for 1 h (re-plating procedure). Cell quantification was assessed using trypan-blue, followed by FACS analysis of unstained cell suspensions to determine the GFP-positive fraction. Alternatively, 0.5 × 10^6^ purified (replated) and unpurified cells were fixed using 0.1% PFA, stained using anti-MEF-PE (Miltenyi Biotec, Germany) (1:11) in 0.5% BSA buffer and normal mouse IgG-PE (Santa Cruz Biotechnology, USA) as a isotype control for 10 min in 2–8°C in darkness. Enhanced GFP-fluorescence and anti-feeder-PE staining were confirmed using fluorescence microscopy immediately prior to FACS analysis (see Figures 3A,B). FACS analysis was performed using FACS ARIA (Becton Dickinson, USA) and analyzed with WinMDI2.8 (Scripps Research Institute, USA).

### MEF implantation

All experiments were performed according to the animal protection guidelines and were approved and registered by the local governmental authorities of North-Rhine Westphalia, Germany. The animals included in this study primarily served as a control group in the previous study (Molcanyi et al., 2009). Adult male Sprague-Dawley rats (250–300 g, age 12–14 weeks, supplied by Harlan, Germany) were intraperitoneally anesthetized with 60 mg/kg body weight pentobarbital. The animals were placed in a stereotactic frame (David Kopf Instruments, USA). The cranial soft tissue was opened and a small craniotomy was drilled, using a 2-mm trephine at calculated coordinates. Five microliters of a cell suspension containing 5 × 10^3^ MEF cells in PBS were injected using a Hamilton needle under stereotactic conditions at the following coordinates: AP −3.4, ML 5.0, and DV −3.2 relative to bregma. The decision, regarding the amount of MEFs to implant (5 × 10^3^) was initially met based on the fluorescent cell-counting assessment of MEF portion, which contaminated the co-culture/cell graft. FACS re-evaluation of MEF contamination under standardized co-culturing conditions of D3-ESC/MEFs in our facility (ranging from approximately 1 to 10% of a standard graft containing 10^5^ cells) approved the MEF amount implemented in the *in vivo* part of the study. MEF grafts were placed into the cortex of eight animals. The control group, which consisted of six animals, received an analogical injection of PBS. Twenty-four hours prior to implantation, the animals received an intraperitoneal injection of cyclosporin A (CsA, 10 mg/kg body weight, Sandimun, Novartis, Germany) as previously described (Molcanyi et al., 2007, 2009). Subsequently, the immunosuppressive drug was administered daily for up to 14 days after implantation. Animals were sacrificed 14 days post-implantation by lethal dose of sodium pentobarbital and transcardially perfused with 200 ml of heparinized PBS followed by 250 ml of 4% paraformaldehyde solution (Merck, Germany). Brains were removed from the skull, post-fixed in 2% paraformaldehyde for 2 days, processed, and embedded in paraffin blocks.

### Histology and immunohistochemistry

Paraffin-embedded brains were cut using a microtome (6 μm coronal sections) and mounted on poly-l-lysine coated glass slides (Biochrom, Germany). Dewaxing and rehydration were performed using subsequent xylene, alcohol, and distilled-water baths. Hematoxylin-eosin (HE) and Nissl stainings were performed according to standard protocols. Alcian-blue staining, which visualizes extracellular substances, such as acidic polysaccharides (e.g., glycosaminoglycans and mucopolysaccharides), was performed according to a standard protocol under pH-controlled conditions. Van Gieson staining, which detects collagen, was performed according to a standard protocol.

Conventional immunohistochemistry was started by blocking of endogenous peroxidase using 1% H_2_O_2_ (Merck, Germany) in methanol (Merck, Germany) for 20 min. The sections were shortly microwaved (1200 W, 1 min.) in a pH6 antigen-retrieval solution (DAKO, Denmark). Non-specific bindings were blocked using 5% NGS in PBS/Triton solution (analogous to immunocytochemistry). Monoclonal anti-CD-68/ED-1 (1:100, Serotec, Germany) in NGS-PBS-Triton solution was applied to sections for 2 h at room temperature. After two PBS-wash steps, the sections were incubated with biotinylated goat anti-mouse antibody (1:100, DAKO, Denmark) for 2 h at room temperature and visualized using streptavidin-horseradish peroxidase/chromogen 3,3′-diaminobenzidine (DAB) systems as recommended by the manufacturer: VECTASTAIN Elite ABC (Vector Laboratories, USA) and (DAKO, Denmark). For fluorescent immunohistochemistry, the sections were blocked with 5% NGS in PBS-Triton, incubated with anti-feeder-PE antibody (1:100 for 2 h at room temperature), and additionally incubated with anti-rat-Alexa555 antibody (1:100 for 2 h at room temperature) to amplify the signal of PE-conjugated antibody (primarily developed for flow-cytometry), followed by DAPI-counterstaining. Adjacent sections, which were incubated with a secondary antibody (omitting the primary antibody) served as negative controls. All specimens were viewed using a conventional/fluorescent Leica DMRB microscope (Leica, Germany) equipped with a 3CCD JVC live-camera (JVC, Japan). Images were captured using Diskus imaging software (Königswinter, Germany). Supplementary data (see Introduction) shows a histological section, which neighbored the section that was previously published but not the identical one. The animal demonstrating post-implantation MEF survival was briefly mentioned but not further analyzed in our previous publication (Molcanyi et al., 2007, 2009).

### Statistics

Immunocytochemistry was performed on *n* = 12 culture dishes of MEF cells and *n* = 4 culture dishes of CGR8 cells (grown on coverslips). Immunohistochemistry was performed in 14 (*n* = 8 grafted, *n* = 6 control) animals. Cell loss during the re-plating procedure and further FACS analysis were assessed using 14 ESC/MEF culture dishes (*n* = 7 untreated and *n* = 7 gelatin-replated). The Kolmogonov–Smirnov-test was performed to evaluate the data distribution. All data sets exhibited a normal distribution. The maxima and minima were designated as individual %-values; all other results are shown as the mean ± standard deviation, if not otherwise stated. We used *t*-tests for group comparison. *P* < 0.05 was considered to be significant. Statistical analyses were performed using IBM SPSS (IBM, USA).

## Results

**Figure 1:**
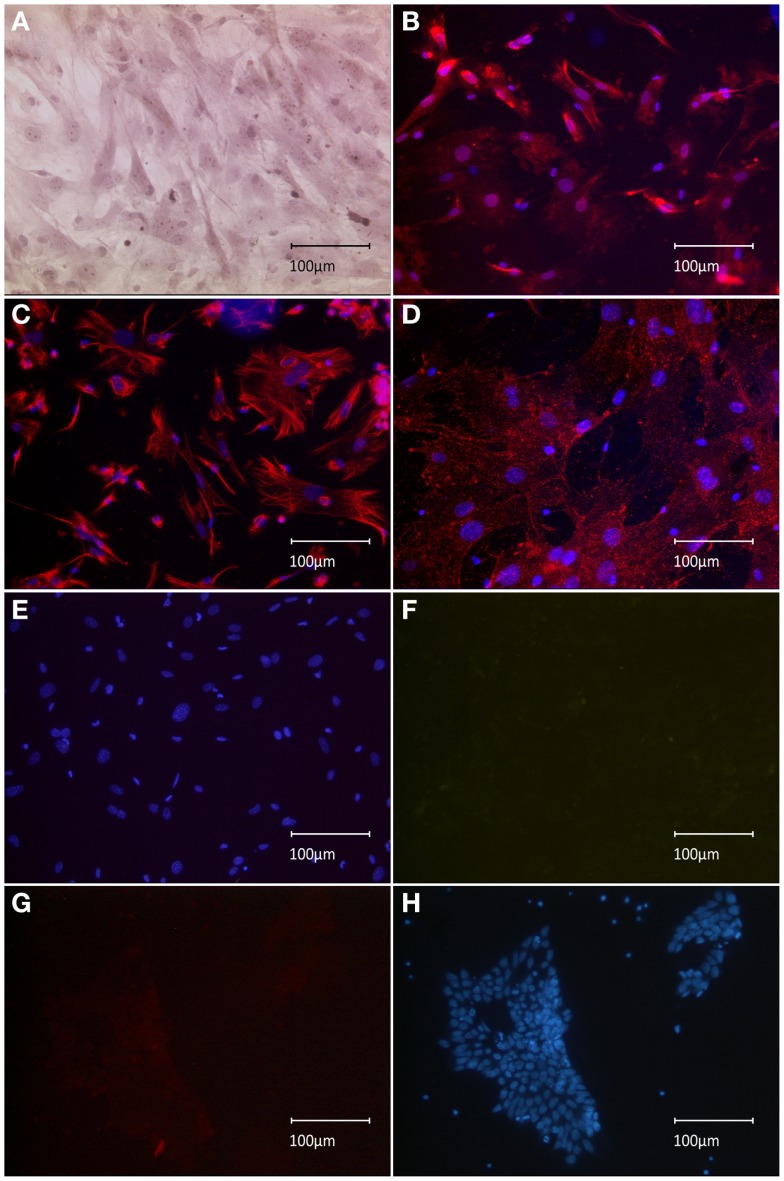
**Immunocytochemical characterization of MEF monolayer *in vitro***. **(A)** HE staining revealed the presence of both spindle-form and planar-shaped cells. **(B)** Anti-nestin-Alexa555 staining is strongly exhibited by spindle-shaped fibroblasts; much weaker signal is associated with planar phenotypes. **(C)** Uniform anti-vimentin-Alexa555 staining of all cells indicates a pure fibroblast monoculture. **(D)** Labeling by anti-feeder-PE antibody, amplified by anti-rat-Alexa555 yielded a uniform staining of all cells exhibiting a granular pattern. **(E)** Control dishes (omitted primary antibody) incubated with secondary antibodies and counterstained by DAPI showed no specific signal in the red emission spectrum and **(F)** no non-specific background autofluorescence when inspected in the green emission channel. **(G)** No specific staining of the CGR8 cell line using the anti-feeder-PE antibody in the red channel, **(F)** the same CGR8 culture area counterstained with DAPI.

To confirm our initial presumption that previously observed nestin+ spindle-shaped cells might have been co-transplanted MEFs, the expression profile of implemented feeder-cell line was characterized. Sub-confluent MEFs manifested both spindle-like and planar phenotypes, as observed using HE staining (Figure 1A). Spindle-shaped cells stained positive for nestin, which supported our initial hypothesis regarding co-transplanted feeders (Figure 1B). Phenotypic characterization of the MEF culture showed vimentin staining of the entire population, including both spindle-shaped and flat cells, indicating a pure fibroblast monoculture (Figure 1C). Furthermore, we successfully tested a novel anti-feeder-PE antibody, which was initially developed for FACS and depletion procedures (Knoebel et al., 2010). Specific antibody binding was determined from granular staining, which covered all cell phenotypes (Figure 1D). Controls, which were counterstained using DAPI (lacking primary antibodies), showed no specific signals in the red emission spectrum and no non-specific autofluorescence in the green channel (Figures 1E,F). An additional negative control, the CGR8 cell line, showed no specific labeling using the anti-feeder antibody (Figures 1G,H).

**Figure 2:**
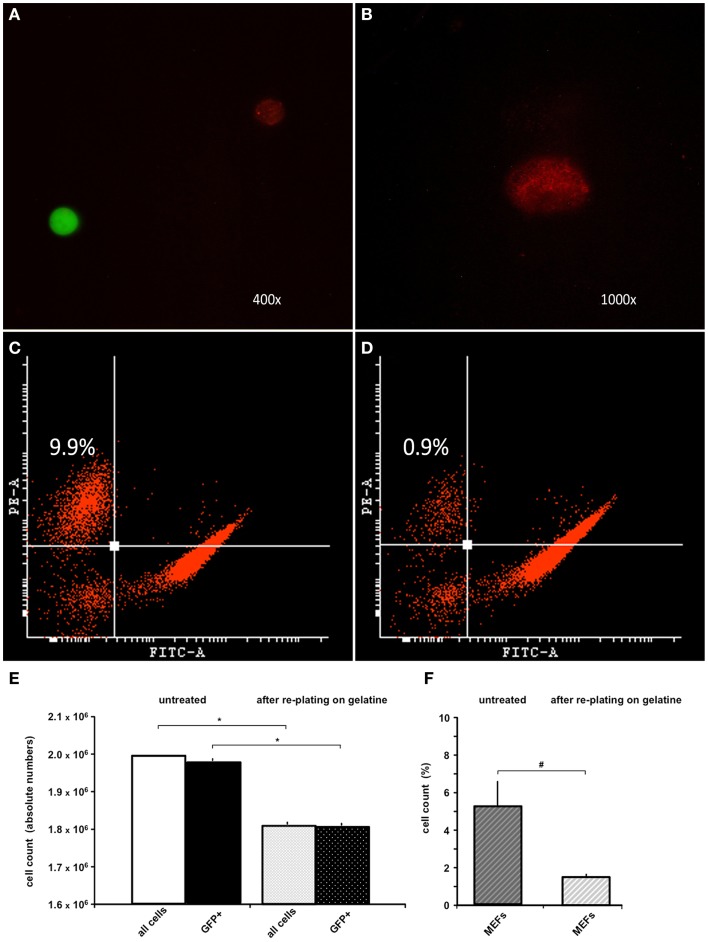
**FACS analysis of trypsinized ESC-MEF co-culture with/without re-plating on a gelatin-coated dish**. **(A)** Confirmation of specific signaling using fluorescent microscopy prior to FACS: GFP-positive ESCs exhibited high-intensity signal in the green emission spectrum, MEFs labeled using the anti-feeder-PE antibody emitted a specific red signal. **(B)** Grainy pattern of the specific anti-feeder-PE labeling, as previously observed in the immunocytochemistry data. **(C)** Trypsinized ESC-MEF cell suspension showed a well-delineated GFP+ cell population depicted on the right side of the plot, with the anti-feeder-PE + MEF- fraction situated in the left upper corner; this particular measurement showed the highest measured value of 9.9% of the overall cell-count. **(D)** Amount of MEFs contaminating the cell suspension decreased to a minimum of 0.9% after re-plating step (Note: both plots show representative maximum and minimum values acquired by FACS assessment of two individual cell-culture dishes, which was further followed by repeated measurements of additional dishes – see next) **(E)** Bar diagram presenting the absolute values (mean ± SD) of *n* = 7 untreated cell suspensions and *n* = 7 after re-plating on gelatin-coated dish, with a total cell loss accounting for approximately 10% of the primary cell suspension. The cell loss was statistically significant, when examined for both the entire cell suspension and GFP+ fraction – marked by *p < 0.001. **(F)** Additional diagram presenting %-mean and ±SD values of FACS-acquired MEF contaminations in untreated versus gelatin-treated dishes, showing a statistically significant reduction of MEF amount – marked as ^#^*p* = 0.011. Despite the reduction, MEF contamination still accounted for 1.4 ± 0.2% of the entire cell suspension.

Because the anti-feeder-antibody was demonstrated to bind specifically to established MEF cultures, we also used this antibody in FACS analysis of the MEF/ESC co-culture. GFP-positive ESCs grown on a MEF monolayer were detached from the dish and incubated using the anti-feeder antibody. Specific signals of the cell suspension (native GFP-fluorescence of ESCs and red-fluorescence of anti-feeder-PE stained MEFs) were examined and confirmed using fluorescent microscopy prior to FACS (Figures 2A,B). Anti-feeder-PE staining exhibited a typical granular pattern, as previously observed (compare with Figure 1D). Analysis of detached cell suspensions showed that the feeder cells accounted for 5.33 ± 2.81% (full range 2.2–9.9%) of the entire cell suspension (Figures 2C,F). Alternatively, cell suspensions were replated on a gelatin-coated dish for 1 h; this is an extra step that allows the feeder-cell fraction to attach. Free-floating cell populations were harvested and assessed by FACS, which yielded a significant reduction in the feeder-cell fraction down to 1.45 ± 0.27% (full range 0.9–1.7%) (*p* = 0.011, *n* = 7, respectively; Figures 2D,F). The re-plating step also led to a reduction in the entire cell suspension (overall decrease in cell counts, compared to untreated dishes), as apparently both MEFs and also ESCs attached to gelatine coating to some extent. The total cell loss and the concomitant loss of GFP+ cell fraction were both significant and was as high as approximately 10% of the initial cell suspension (*p* < 0.001, *n* = 7, respectively; Figure 2E). The effect of single re-plating was examined in this study, as some authors previously implemented this procedure to reduce the number of contaminating MEFs, prior to the transplantation (see Discussion). Currently assessed FACS-values of %-MEF contamination under standardized co-culturing conditions of D3-ESC/MEFs in our facility highlighted the presence of a considerable MEF-fraction in both untreated/gelatin-treated cultures and justified the cell amount used in the *in vivo* part of this study (see Materials and Methods).

**Figure 3:**
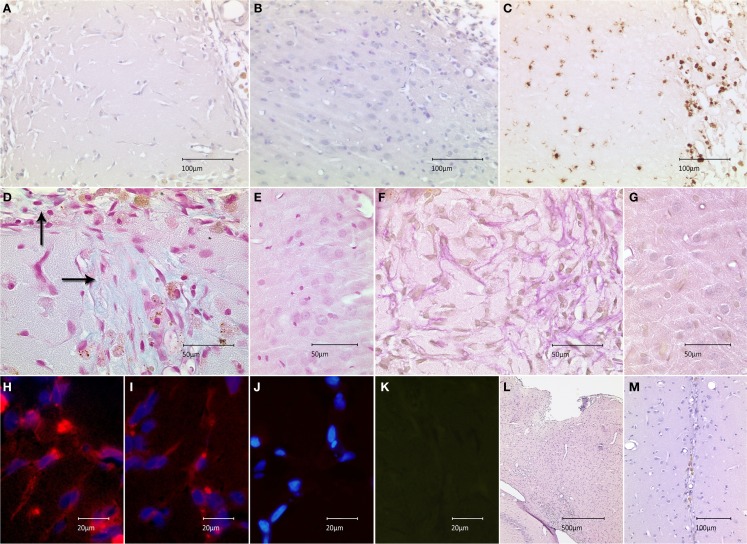
**Survival of feeder cells and their metabolic activity after implantation into healthy rat brain is shown**. **(A)** Spindle-shape cells at close proximity of implantation site in HE-stained section with marginal infiltration of hemosiderin-laden cells compared to **(B)** adjacent cortex. **(C)** Infiltrating cells stained positive for the macrophage marker CD68-DAB; the staining shows abundant populations at the implantation site and minor invasion of the adjacent cortex. **(D)** Alcian-blue staining at the implantation site, indicating the presence of acidic polysaccharides (extracellular matrix) with **(E)** no signs of specific blue signal in the healthy cortex (control). **(F)** Abundant red staining, secundum Van Gieson, indicates the presence of collagenous extracellular matrix with **(G)** missing collagen expression in the healthy cortex. **(H,I)** Anti-feeder-PE staining demonstrating spindle-shaped cells to be implanted MEFs; with no specific red signal in **(J)** control section (omitted first antibody, counterstained by DAPI) and **(K)** no interfering autofluorescence as examined in the green emission channel. **(L)** Cortical discontinuity and hypercellularity at the site of former implantation in Nissl-stained animals lacking surviving fibroblasts. **(M)** Needle-track with some hemosiderin-laden cells in control animals receiving PBS injection.

To examine the translational effect of inactivated MEFs, we transplanted 5 × 10^3^ MEFs into the cortices of healthy rat brains and evaluated their survival and interaction with surrounding microenvironment *in vivo*. The transplantation procedure was successfully performed in all animals; eight rats receiving MEFs and six control animals receiving a PBS injection. All animals survived the observation period of 2 weeks. Next, the animals were sacrificed and histologically examined. The site of the former graft implantation could easily be identified in all HE- and Nissl-stained brains (see below and description of Figure 3). In seven grafted animals, no fibroblast-resembling cells could morphologically be discerned, most likely due to macrophage-mediated clearance. In one animal, spindle-shaped cells were found in close proximity to the transplantation site, which was clearly delineated from the neighboring cortex (Figures 3A,B). The transplantation site exhibited a cortical discontinuity, which was invaded by round cells of variable sizes, many of which carried hemosiderin deposits. Considerable invasion could also be observed in the adjacent cortex. Anti-CD68 staining demonstrated that these cells were macrophages, which were most likely responsible for scavenging of the implanted graft (Figure 3C). As the texture of transplantation site differed from healthy cortical tissue, we tested for the presence of extracellular matrix as a potential by-product of the implanted feeder cells. Alcian-blue staining confirmed the presence of acidic polysaccharides inside and at the margin of the transplantation site (no blue signal detected in the healthy cortex) (Figures 3D,E). An abundant presence of collagen was confirmed using van Gieson staining at the site of transplantation (no red staining detected in the healthy cortex) (Figures 3F,G). Both stainings embodied an indirect proof of survival and metabolic activity of the implanted feeder cells. Labeling of the sections of this animal using an anti-feeder-PE antibody resulted in specific signals that morphologically resembled spindle-shaped cells within the red emission spectrum with no specific red signal in the control slides and no interfering autofluorescence (as additionally inspected in the green emission channel) (Figures 3H–K). The implantation sites were identified in all HE and Nissl-stained brains, based on the presence of cortical discontinuity and hypercellularity (in grafted brains) and needle-track (in control animals) (Figures 3L,M). The labeling of these brains, primarily exhibiting no morphological characteristics of MEF survival, using anti-feeder-PE antibody showed no specific signaling (data not shown). These findings represent the mid-term survival and metabolic activity of feeder cells after transplantation into a healthy rat brain, as a proof of principle.

## Discussion

The results of the present study demonstrate a feeder-cell layer consisting of MEFs to be a source of impurity, which may interfere with translational downstream ESC applications. Characterization of MEFs *in vitro* and the degree of ESC-graft contamination were evaluated using immunocytochemistry and FACS analysis. Implantation of feeder cells into healthy rat brains was performed to evaluate the mid-term survival and metabolic activity of grafted MEFs *in vivo*.

In the past two decades, various cell lines have been used in a number of translational studies, which focus on the experimental therapy of stroke and other central nervous system disorders. Many of the ESCs or precursor cells utilized in these studies required an initial co-culturing with feeder cells for non-differentiated growth, self-renewal, and/or expression of some particular characteristics (Bjorklund et al., 2002; Barberi et al., 2003; Schouten et al., 2004; Burns et al., 2009; Locatelli et al., 2009; Kawai et al., 2010; Richardson et al., 2010; Benchoua and Onteniente, 2011; Rhee et al., 2011; Oki et al., 2012; Polentes et al., 2012; Jensen et al., 2013; Cattaneo and Bonfanti, 2014). However, for the use of ESCs in translational applications or tissue engineering, feeder cells have to be considered as contaminations that might interfere not just with the analysis of experimental data but also with the integration and function of transplanted cells *in vivo* (Schneider et al., 2008). Potential adverse effects of the contaminating feeder cells have been proposed to account for the discrepant results in pre/clinical studies observed by different research groups (Pereira et al., 2011). The depletion of feeder cells from stem cells prior to implantation has rarely been discussed in experimental cell replacement studies, although different methods of reducing the feeder-cell content have previously been described as early as in 1980s. Most of these feeder-reducing methods were based on the different adhesive characteristics of stem and feeder cells, i.e., the preferential adhesion of MEFs to uncoated culture plates (alternatively coupled to solid-phase immunoadsorption) or the weaning off feeders over several passages (Edwards et al., 1980; Halaban and Alfano, 1984; Paraskeva et al., 1985; Linge et al., 1989; Knoebel et al., 2010; Jensen et al., 2013).

However, these methods do not achieve a complete removal of MEFs in one single step and are associated with a concomitant loss of ESC populations. Li et al. reported MEF contaminations as high as 17.5–25% despite two rounds of re-plating on uncoated dishes (Li et al., 2008). In contrast, other authors considered a single re-plating or even a simple trypsinization to be appropriate to eliminate MEF contamination prior to the transplantation procedure (Shintani et al., 2008; Kawai et al., 2010). Generally, MEF contamination is thought to account for approximately 10% of the primary cell suspension, which is consistent with our findings (Knoebel et al., 2010). The degree of contamination is variable, depending on the cell line used and facility-based culturing protocols. Considerable advances were achieved after the re-plating technique was amended using additional gradient separation. Utilizing this method, ESCs were enriched to purity >99% with a recovery rate higher than 90% (Li et al., 2008). Our purification step, implementing a gelatin-coated culture dish, also resulted in a high MEF adhesion and reduction of contamination to 1.45 ± 0.27%, in contrast to higher contaminations, which were reported when using uncoated dishes (Knoebel et al., 2010). However, in the case of *in vivo* applications, even a minor contamination of large batch preparations can subsequently translate into a substantial cell count and associated complications (Li et al., 2008; Pereira et al., 2011).

Until recently, cell-sorting technologies (such as MACS Cell Separation) could not be utilized for feeder depletion due to the lack of a pan-fibroblast surface marker, which is common to all feeder strains. However, a novel mEF-SK4 antibody, which specifically docks to all tested fibroblast types, was newly developed and coupled to paramagnetic particles (Feeder Removal MicroBeads) for subsequent MACS cell separation of feeders from ESCs. This technology was shown to be a superior system for the efficient selection of highly purified stem cell populations, which contain <0.15% remaining MEFs (Knoebel et al., 2010). PE-conjugated mEF-SK4 antibody (alternatively amplified by Alexa555 for immunocyto- and histochemistry) was also successfully used to label the feeder cells in our study (Figures 1D, 2A,B, 3H,I). Alternatively, a complete feeder-free purification of stem cells was also achieved using an automated cell selection system, which aimed at the aspiration of distinct stem cell colonies. In this previously established method, we showed that the early complete “freeing” of stem cell colonies from feeder cells did not interfere with subsequent differentiation processes *in vitro* (Schneider et al., 2008). Thus, the complete withdrawal of feeder cells should be considered for all downstream translational approaches, because of potentially detrimental effects of contaminating feeder cells (Li et al., 2008; Schneider et al., 2008; Pereira et al., 2011).

It is widely accepted that detrimental effects may occur due to the release of a variety of humoral factors, cell–cell interactions at transplantation site (with both grafted ESCs and surrounding host cells), or via activation of the immune response of the host–environment. Various interactions between different cell types and the brain microenvironment were reported in several studies (Bentz et al., 2006, 2007, 2010; Molcanyi et al., 2007). We previously showed that incubation of ESCs with brain extract *in vitro* resulted in the release of neurotrophic factors, which was accompanied by the considerable co-production of these neurotrophins by inactivated co-cultured MEFs (Bentz et al., 2007). The metabolic potential of inactivated feeder cells is not surprising as MEFs are expected to produce humoral factors, which maintain the characteristics of co-cultured ESCs. Contaminating MEFs are similarly suspected to continually secrete anti-differentiation factors *in vivo*, which exert an effect on the local microenvironment after co-transplantation (Li et al., 2008). The effect of this phenomenon on tumorigenesis after cell grafting in experimental models of stroke and other cerebral disorders has remained unresolved (Molcanyi et al., 2009). Moreover, our current study demonstrated the release of MEF-associated extracellular matrix (otherwise, not present in healthy brain tissue), which may negatively affect the local microenvironment, as well (Figures 3D,F).

In this study, we observed a pronounced immune reaction at the site of MEF implantation despite administered immunosuppression (Figure 3C). Previously, the cellular immune response was shown to be responsible for scavenging the stem cell grafts implanted into the central nervous system (Li et al., 2005; Molcanyi et al., 2007). Immune system activation has been observed and attributed to different mechanisms, such as rejection, the removal of necrotic and/or apoptotic cells, or the combination of trauma and transplantation stimulus (Olanow et al., 2003; Li et al., 2005; Molcanyi et al., 2007, 2013; Pereira et al., 2011). Immune cell infiltration observed in the current study was very likely due to macrophage activation (first line defense) as a response to a local stimulus (needle injury and grafting of heterotopic cell suspension), rather than a specific host-versus-graft rejection. However, this issue is not completely resolved yet and the authors are planning to examine the immune response in all previously grafted brains (injured and healthy ones) in contrast to the brains receiving different control media (PBS versus feeder cells). In the light of current findings, additional amplification of immune response by co-transplanted feeder cells appears to be likely. This assumption is consistent with the observations of other authors (Pereira et al., 2011) who proposed that implanted fibroblasts activate immune cascades, resulting in detrimental effects. Pereira et al. showed that umbilical cord-derived mesenchymal stem cells induced potent neuroprotection in a rat model of Parkinson’s disease. However, transplantation of fibroblast-contaminated grafts reversed the therapeutic efficacy and caused harmful effects, such as exacerbation of neurodegeneration and motor deficits. Surviving fibroblasts were observed as late as 3 weeks after engraftment into the rat striatum in their study (Pereira et al., 2011), which confirmed our observations.

In the beginning of the cell-therapy era, pluripotent cells maintained on feeder layers were thought to engraft, differentiate, and replace lost cells in the damaged target tissue. Negative effects, such as tumor formation resulted in a paradigm shift toward the use of precursor cells, as the pre-differentiation was shown to circumvent the threat of tumorigenesis (Benchoua and Onteniente, 2011). In addition, feeder-based cell lines were shown to be contaminated by animal proteins, which interfered with implementation in clinical use (Bardor et al., 2005; Klimanskaya et al., 2005; Lanctot et al., 2007). This resulted in the establishment of feeder-free and subsequently entirely xeno-free culture conditions (Klimanskaya et al., 2005; Marinho et al., 2013). However, many cell lines (both embryonic and induced pluripotent stem cells) still required the presence of feeder cells such as MEFs, MSCs, or HDFs, at least in a specific phase of the culture protocol, e.g., for initial propagation and expansion (Klimanskaya et al., 2005; Willmann et al., 2013). Subsequent differentiation into neural phenotypes was temporarily performed using different feeder or stromal cells (e.g., PA6, MS5, MS5SHH, S2, Sertoli cells) (Perrier et al., 2004; Saporta et al., 2004; Benchoua and Onteniente, 2011; Kim and Park, 2011; Rhee et al., 2011). Shintani et al., who were aware of potential contamination, developed a differentiation protocol using bone marrow stromal cells (BMSC), resulting in the generation of functional dopaminergic neurons. Thus, the contamination of the neural graft by co-cultured autologous BMSCs (particularly, if implemented in a clinical testing) presents a risk, which was considerably lower than that of xenogeneic feeders (Shintani et al., 2008). Contemporary differentiation protocols translocate once feeder-initiated stem cells onto coated dishes for terminal differentiation in the absence of a feeder layer (Barberi et al., 2003; Perrier et al., 2004; Dubois-Dauphin et al., 2010; Kim and Park, 2011; Rhee et al., 2011). Further advancements have moved toward the culturing and differentiation of stem cells in completely feeder-free conditions (Cooper et al., 2010). The conclusions of our study highlight the necessity of this trend; conversely, the trend also presents a major limitation of our study, as we currently do not expect pluripotent stem cells or feeder-layer-based precursors to be considered for translational applications. Another limitation of our study is the observation of MEF-survival, which is restricted to one animal. However, the presence of MEFs has been proven by directly using a specific anti-feeder-antibody and indirectly by demonstrating the formation of MEF-associated extracellular substances. Consistent with our observations, analogous data were obtained by Perreira et al., who detected fibroblasts (using a species-specific antibody) surviving up to 3 weeks after transplantation in rat brains (Pereira et al., 2011).

## Conclusion

The majority of previously implemented embryonic and induced pluripotent cell lines required the presence of an additional feeder-cell layer at a specific phase of the culturing protocol. It is known that feeder cells present a potential source of impurity, e.g., in the form of feeder-derived xeno-proteins. In this study, we analyzed the level of direct MEF contamination in ESC preparations and their engraftment *in vivo*. Despite the re-plating procedure, the residual impurity *in vitro* was still evident. Our observations confirm MEFs to impede the transplantation strategies, as they are able to survive a mid-term period after grafting and to produce extracellular substance *in vivo*. Presented data clearly support the current trend aiming for feeder-free technologies and provides critical insight into MEF effects with respect to graft fate, cell commitment, and graft–host interaction. These observations should be considered when interpreting a broad spectrum of previously published studies of this field.

## Author Contributions

Marek Molcanyi, Ute Schäfer, Peter Riess, Jürgen Hescheler, and Bert Bosche conceived the study. Marek Molcanyi, Narges Zare Mehrjardi, Nadia Nabil Haj-Yasein, Michael Brockmann, Marina Penner, Peter Riess, Bernhard Rieger, Tobias Hannes, and Bert Bosche performed the experiments and data analyses. Marek Molcanyi, Ute Schäfer, Michael Brockmann, Peter Riess, Clemens Reinshagen, Jürgen Hescheler, and Bert Bosche wrote the manuscript.

## Conflict of Interest Statement

The Guest Associate Editor Thorsten Doeppner declares that, despite being affiliated to the same institution as author Bert Bosche, the review process was handled objectively and no conflict of interest exists. The authors declare that the research was conducted in the absence of any commercial or financial relationships that could be construed as a potential conflict of interest.

## Supplementary Material

The Supplementary Material for this article can be found online at http://www.frontiersin.org/Journal/10.3389/fncel.2014.00257/abstract
