# Supplementary material for: Impurity of Stem Cell Graft by Murine Embryonic Fibroblasts – Implications for Cell-Based Therapy of the Central Nervous System
Source: Front Cell Neurosci. 2014 Sep 5;8:257. doi: 10.3389/fncel.2014.00257 (PMC4155790; doi:10.3389/fncel.2014.00257)
Supplement: Supplementary file 1 [file Presentation1.PDF]

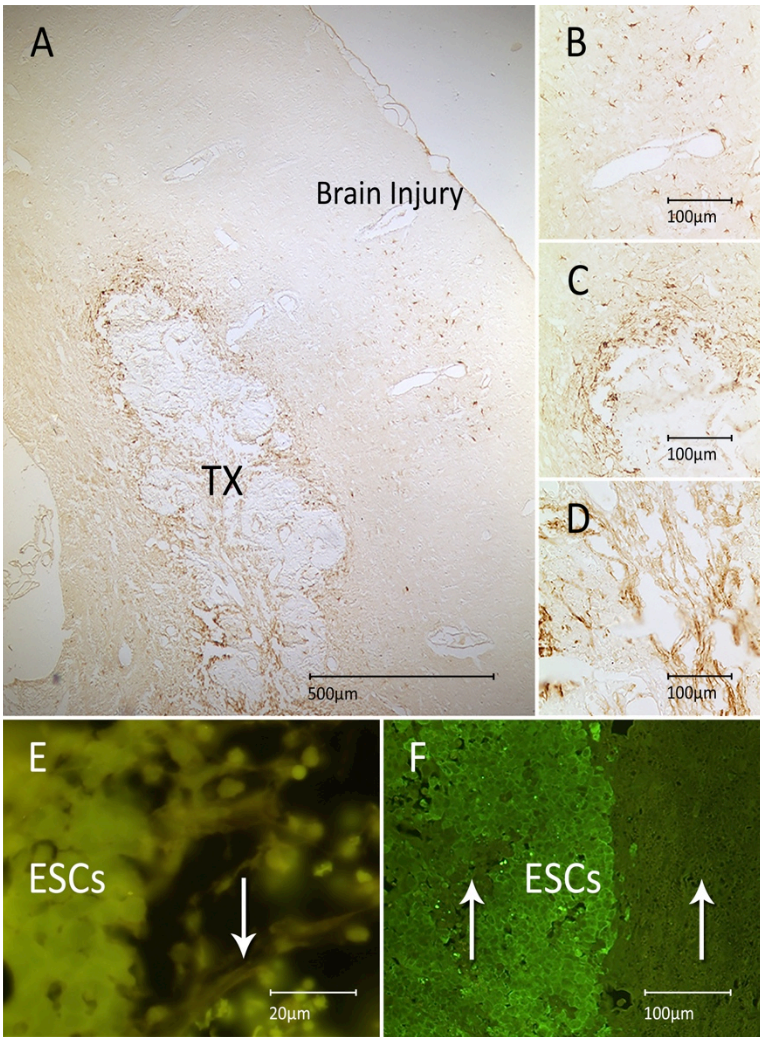

SUPPLEMENTARY FIGURE | Presence of nestin+ cells at the site of trauma and implantation. Implantation of GFP-transfected ESCs (D3 cell line) in a close proximity of trauma site performed 72 hrs. after a moderate lateral fluid-percussion injury. Histological examination performed 5 days postimplantation. (A) Injured cortical area marked as ”Brain Injury“, transplanted ESC-cluster marked by ”TX“ (section stained by anti-nestin-DAB, implanted ESCs remain unstained). Nestin+ cells were brown in color and marked different populations: **(B)** trauma-activated astrocytes in the injured cortex **(C)** activated astrocytes around the implanted cluster **(D)** spindle-shaped cellular elements exhibiting variably strong nestin-positivity. These cell populations were shown to be GFP-negative in double-labeling confocal analysis, using advanced fluorescent techniques (including converse staining with anti-GFP-Cy3&anti-nestin-FITC) as previously described **(E)** Spindle-shaped elements, marked by an arrow, emit no specific GFP-signal, when viewed in the green channel (evaluating native GFP-fluorescence) **(F)** Their signal is analogous to that of GFP-negative cells inside the implanted graft (left arrow) or the weak background fluorescence surrounding brain tissue (right arrow); technical note: ESC-signal was amplified using anti-GFP-FITC in this section. For further methodological details, see Molcanyi et al. 2007 doi: 10.1089/neu.2006.0180.
